# Price Movement Prediction of Cryptocurrencies Using Sentiment Analysis and Machine Learning

**DOI:** 10.3390/e21060589

**Published:** 2019-06-14

**Authors:** Franco Valencia, Alfonso Gómez-Espinosa, Benjamín Valdés-Aguirre

**Affiliations:** Tecnologico de Monterrey, Escuela de Ingeniería y Ciencias, Ave. Epigmenio González 500, Fracc. San Pablo, Querétaro 76130, Mexico

**Keywords:** price movement, cryptocurrencies, sentiment analysis, machine learning

## Abstract

Cryptocurrencies are becoming increasingly relevant in the financial world and can be considered as an emerging market. The low barrier of entry and high data availability of the cryptocurrency market makes it an excellent subject of study, from which it is possible to derive insights into the behavior of markets through the application of sentiment analysis and machine learning techniques for the challenging task of stock market prediction. While there have been some previous studies, most of them have focused exclusively on the behavior of *Bitcoin*. In this paper, we propose the usage of common machine learning tools and available social media data for predicting the price movement of the *Bitcoin, Ethereum, Ripple* and *Litecoin* cryptocurrency market movements. We compare the utilization of neural networks (NN), support vector machines (SVM) and random forest (RF) while using elements from Twitter and market data as input features. The results show that it is possible to predict cryptocurrency markets using machine learning and sentiment analysis, where Twitter data by itself could be used to predict certain cryptocurrencies and that NN outperform the other models.

## 1. Introduction

Although there are some studies that deal with both the task of predicting stock market price movements, as well as the development of profitable trading strategies based on those predictions, it is important to verify the applicability of such studies in new and emerging markets; in particular the cryptocurrency market.

This market is characterized by high volatility, no closed trading periods, relatively smaller capitalization, and high market data availability [1]. The financial feasibility of the cryptocurrency market in relation to other markets has been documented [2,3,4] and the algorithms upon which the cryptocurrencies operate have been validated in other fields as well [5,6]. The cryptocurrency market seems to behave independently from the other financial markets [2], but there is a strongly influenced by Asian economies [3]. Part of the appeal behind this market is that the technology used for mining cryptocurrency provides feasible alternative to more traditional markets such as gold [4].

These characteristics have attracted a considerable amount of capital, however up to now there are few studies that have attempted to create profitable trading strategies in the cryptocurrency market [7,8].

Another point of interest in the cryptocurrency market is the large-scale of available public sentiment data, particularly from social networks. This data can presumably be used to infer future human behavior, and therefore could be used to develop advantageous trading strategies [9,10] as has been shown in recent attempts to detect speculative bubbles in the cryptocurrency market using sentiment analysis [11].

Stock market prediction has always been regarded as a challenging task that has attracted attention from both academia and investors [12,13]. For example [12] observed that aggregate stock market returns could not be predicted from Baker and Wurgler Sentiment index (SBW) nor Huang Partial-Least-Squares Sentiment index (SPLS), which aggregates information from six proxies. Whereas [13] found that in Bitcoin market, as opposed to returns, prices incorporate and exhibit chaotic dynamics and uncertainty level in returns significantly increased during the high-price regime period.

The complexity of the task can be attributed to the multiple factors and uncertainties that interact in the markets including economic and political conditions, as well as human behavior. Being able to consistently predict the market price movements is quite difficult, but not impossible. According to academic research, movements in the market prices are not random, but behave in a highly non-linear and dynamic way. Previous studies have also shown that it is not necessary to be able to foretell the exact value of the future price in order to make profit in financial predictions. In reality, predicting the market direction as compared to its value can result in higher profits [14].

Over the past decades, artificial intelligence and machine learning techniques have been used to predict the stock market. Neural networks (NNs), support vector machines (SVMs) and random forests (RFs) have been the most widely used techniques. Derived from NNs come deep learning approaches, which have been used to forecast the price of Bitcoin, Digital Cash and Ripple [15], and recurrent neural networks used to predict the direction-of-change of the market in the case of the NASDAQ composite index [16].

Most successful models treat stock market prediction not as a regression problem as one could expect, but as a classification problem. Significant progress has been made in the prediction of the price movement direction of the Standard & Poor’s 500 stock index futures on a daily basis [17,18,19].

For NNs, there have been multiple studies that have shown the utility of BP algorithms in stock market prediction problems [20,21], and how easily BP algorithms can outperform even the best regression models for this task [22].

SVMs are also used because of their effectiveness in high dimensional spaces and that unlike NNs, SVMs are resistant to over-fitting. These features have made SVMs a popular choice for financial forecasting and stock market prediction [23,24]. Some studies have even found that SVMs outperform other classification methods and as such are the best model for forecasting market movement directions [25]. However others have found that BP or SVMs superiority over each other is dependent on the market [26].

A study comparing NNs, SVMs, RFs and naive-Bayes performance for stock price index movement in Indian stock markets, found that RFs outperformed the other models, when the model was trained with ten technical parameters that were presented as continuous values [27]. Later in another study [28] it was suggested that a Random Forest Classifier did indeed outperform other models and algorithms found in the literature.

A more novel approach utilizes social signals and sentiment analysis for the prediction of trading volumes and the prices of individual stocks [29]. Sentiment in social networks, particularly from Twitter, can be used to predict movements in stock indices [9]. While there is no evidence that predictions based on sentiment produce significant returns on stock trading [30], a study was able to obtain a trading strategy based on social media sentiment for the Bitcoin cryptocurrency [8]. Another study expanded the amount of research on alternative cryptocurrencies and proposed a method to predict fluctuations in the prices of the *Bitcoin, Ethereum* and *Ripple* cryptocurrencies using sentimental analysis [31].

The results of these previous studies go in line with the latest findings of a [32]. This study proved that these hypothesized medium-term and short-term relationships between online factors and market events, not only exist, but that they are strengthened during bubble-like financial series.

While cryptocurrencies are a very interesting concept from an economic perspective, more research on their behavior is necessary to determine their viability as an alternative medium of exchange. We hypothesize that its market price is determined by speculation rather by its intrinsic value as currencies. While this hypothesis can not be proven in a single study, we aim to contribute to the research in the area.

In this paper, we extend the application of financial time series forecasting with machine learning and sentiment analysis techniques to alternative cryptocurrency markets. In doing so we expect to show the potential of easily available machine learning tools for exploring the relationships between online factors and cryptocurrency prices.

While doing this, we compare three prediction models: NNs, SVMs and RFs by applying them to four different cryptocurrencies: Bitcoin, Ethereum, Ripple and Litecoin. These cryptocurrencies where selected because at the time, they had the highest market capitalization. We use three approaches for input to these models. The first approach trains the model exclusively with social data, the second trains the model exclusively with market data and the third combines both *social* and market data for training. Then we evaluate the performance of each prediction model, and test whether social media sentiment predicts the market price movements for the cryptocurrency in question.

The rest of this paper is arranged as follows: In Section 2, we give a general introduction to the data, sentiment analysis and machine learning. In Section 3 we present the obtained results with their interpretation. Conclusions and disclaimers are in Section 4.

## 2. Materials and Methods

### 2.1. Market Data

Historical market data was obtained from the top performing 65 cryptocurrency exchanges. The data was fetched from *cryptocompare.com* public API, which allows requesting up to 80 days of historical data from any tradable cryptocurrency for free and the complete historical data can be obtained by request. The data obtained can be requested with either an hourly or daily granularity and contains the opening price, highest price, lowest price, closing price and transaction volume for each time-step.

### 2.2. Social Data

Social data was obtained in the form of raw tweets from Twitter. Tweets were selected by applying the following inclusion criteria:

1. Has been created during the time period the study takes place: previous tweets are not taken into account even when they may be influencing current behavior, as such analysis is outside the scope of this study.
2. Contained the name (i.e., bitcoin) or the ticker symbol (i.e., btc) of one of the analyzed currencies in either its text fields or tags: this gives a high degree of confidence that the tweet is at least related to one of the cryptocurrencies in question.
3. Is written in English: Being dictionary based, our sentiment analysis tool only works with the English language.
4. Is not duplicated: while re-tweets were allowed as this may signal a sentimental trend, duplicated tweets not taken in consideration as this type of activity is mainly displayed by bot accounts.

While the prices of cryptocurrencies may affect each other, we determined that tweets from another cryptocurrency would be less significant than tweets regarding the cryptocurrency that was being analyzed. In order to keep the Twitter data unique for each cryptocurrency, once collected, tweets were split according to what cryptocurrency they belonged to. In case the tweet belonged to more than a single currency, it was added to all the currencies its keywords matched.

Because of the lack of historical data from the Twitter API, tweets had to be collected on a daily basis. This was done by fetching tweets from the Twitter streaming API and saving them in a time-series database.

Averaging 345,000 tweets per day, at the end of the collection period a total of 20,789,572 tweets were obtained.

### 2.3. Sentiment Analysis

Sentiment was measured by applying Valence Sentiment Analysis to the text of the cryptocurrency related *tweets*. Valence quantifies the degree of pleasure or displeasure of an emotional experience.

For the task we used valence aware dictionary and sentiment reasoner (VADER). A sentiment analysis method, specifically designed for social media context. VADER was created from a gold standard sentiment lexicon, that is valence-based and human curated [33]. We selected VADER for multiple reasons: (i) it is an open source tool; (ii) it is human-validated, and specifically attuned for Twitter content; and (iii) it performed extremely well for our purposes in independent benchmarks [34]. The result of applying VADER to a tweet text is a vector with a normalized value for the scores: positive sentiment, neutral sentiment, negative sentiment and compound sentiment.

Most work performed on sentiment analysis for financial markets focuses only on the dimensions of valence, mood or calmness, often overlooking the phenomenon of polarization of opinions. For this reason, in a similar fashion as done previously [8], we calculated a polarization score for each hour of data by applying the geometric mean of the average of the positive sentiment and the negative sentiment of all the tweets that are in the time-step with the intention of using the polarization score as a complementary dimension to emotional valence.

### 2.4. Feature Vectors

A system was set up to gather all collected data from the different data sources, for the creation of a single data set that includes both market and social data. Thus, given the market data and social signals, a feature vector *V* for a certain time period *t* is defined as:

(1) $$V\left(t\right)=\left[\begin{array}{c} neu,\\ norm,\\ neg,\\ pos,\\ pol,\\ close,\\ high,\\ low,\\ open,\\ volumeto \end{array}\right]$$

where,

neu is the average of neutral sentiments defined as $\frac{\sum_{i=1}^{n}t_{neu}}{n}$
neg is the average of negative sentiments defined as $\frac{\sum_{i=1}^{n}t_{neg}}{n}$
norm is the sum of the valence scores of each word defined as $\frac{\sum_{i=1}^{n}t_{norm}}{n}$
pos is the average of positive sentiments $\frac{\sum_{i=1}^{n}t_{pos}}{n}$
pol is the geometric mean of pos and neg defined as $\sqrt{V_{pos}V_{neg}}$
*close* is the closing price in the time period
*high* is the highest price in the time period
*low* is the lowest price in the time period
*open* is the opening price in the time period
*volumeto* is the trading volume for the time period

Having $t_{neu},t_{neg},t_{pos}$ and $t_{norm}$, the VADER calculated scores for each tweet, and *n* for all the tweets that comprised the time period for a certain cryptocurrency.

The target Z(t) is defined as a binary classification with a value of 1 or −1. That represents whether there was an increase or a decrease in price between two time periods. An increment in the closing price between V(t) and V(t+1) would have a Z(t) value of 1. A decrement in the closing price between V(t) and V(t+1) would have a Z(t) value of −1.

The selection of this target was based on the previous knowledge that it was enough to know the direction of the market in order to obtain profit from a prediction [10], as it was previously stated in related research.

### 2.5. Multi-Layer Perceptron

Multi-layer perceptrons (MLPs) are a type of NN that consists of at least three layers of nodes. MLPs may use back propagation and supervised learning for training. As such, they belong to the NN class of Back Propagation (BP). An MLP function can be simply stated as $F\left(\right)=R^{m}->R^{o}$ where *m* is the dimension size of the feature vector and *o* is the dimension size of the target.

How it differs from logistic regression is that it supports the existence of one or more non-lineal layers. The first layer consists of a set of inputs $x_{i}|x_{1},x_{2},\ldots ,x_{m}$ that represent the input features and are connected to the first layer of neurons, known as the input layer. Neurons from the hidden layers apply a lineal summation function $w_{1}x_{1}+w_{2}x_{2}+\cdots +w_{m}x_{m}$ followed by a non-linear activation function to the values of the previous layers. The output layer transforms the values received from the last hidden layer into outputs.

For the usage of any type of NN, it was required to design its architecture. This implies the selection of the number of layers for each type as well as the number of nodes in each of these layers. In order to prevent over-fitting in our NN model, we applied the following heuristic, derived from several assertions and formulas from [35] to calculate $N_{h}$, the upper bound on the number of hidden layers.

(2) $$N_{h}=\frac{N_{S}}{\left(\alpha *\left(N_{i}+N_{o}\right)\right)}$$

$N_{s}$ represents the number of samples in the training data set, α is defined as an arbitrary scaling factor which usually ranges from 5 to 10, $N_{i}$ is the number of input neurons and $N_{o}$ is the number of output neurons.

### 2.6. Support Vector Machines

SVM is a supervised learning algorithm that constructs a hyper-plane or set of hyper-planes, in a high or infinite dimensional space, by the use of a kernel function. SVMs seek to maximize the distance of the hyper-plane from the nearest training examples, by obtaining the training examples that are the closest to the maximum margin hyper-plane which are denominated support vectors. SVMs can be used for classification or regression problems, where the SVM transforms the inputs into a high-dimensional feature space by using a kernel function. The decision function is:

(3) $$y=sgn(\sum_{i=1}^{n}y_{i}\alpha_{i}K\left(x_{i},x\right)+\rho )$$

where *y* is the classification label (1 or −1), *n* is the number of the training vectors, α is a Lagrange multiplier, $K(x_{i},x)$ is the Kernel function and ρ is the intercept for the maximum margin decision boundary.

### 2.7. Random Forests

RFs are meta estimators that fit a number of decision trees on various sub-samples of the data set. RFs use an ensemble approach, combining tree predictors where each tree depends on the values of a random vector with a uniform distribution for all the trees in the forest.

Just as other models, RFs can be applied for classification, using decision tree classifiers. RFs control predictive accuracy and over-fitting by averaging the predictions of each decision tree.

### 2.8. Training

The data set consisted of a time series of market and Twitter data. For training and testing, the data set was divided in a 70–30 split where 70% of the data is reserved for training and 30% is used for testing.

## 3. Results

### 3.1. Setup

The data used for this study was obtained from the sources mentioned in the previous section. We collected 60 days of data from 16 February 2018 to 21 April 2018 as shown in Table 1. The market data had one hour granularity, and the twitter data was processed as previously mentioned to fit this granularity.

A total of 5760 data points where collected, split evenly at 1440 for each cryptocurrency. Since we were performing daily predictions, points were grouped by day for obtaining price movements as shown in Table 2.

We used our prediction models MLPs, SVMs and RFs to foretell the daily market movements of Bitcoin, Ethereum, Ripple and Litecoin. For each cryptocurrency, we compared the performance of the model when using different subsets of the previously defined feature vector V(t). Twitter data was comprised of the V(t) elements *neu, norm, pos* and *pol* while Market data of *close, high, low, open* and *volumeto*. Features where standardized by removing the mean and scaling to unit variance.

All models were implemented using the sci-kit-learn library. Sci-kit-learn provides a toolbox with state-of-the-art models that have a good performance and are versatile. Sci-kit models have a wide range of parameters for each model, with MLP having 21, SVM 14 and RFC 17. Since library already provides excellent default values for the parameters of a model, we only mention the parameters that were fine tuned for this paper. The selection of these fine tuned parameters were selected based on previous work, or because during experimentation they provided more accurate results with the training data.

For our MLP model we selected a hyperbolic tangent activation function because of its popularity and good performance. The default solver, “adam”, a stochastic grading-based optimizer, was utilized with a L2 penalty of 0.0002.

In each experiment, we trained 50 MLPs and results from the best performing networks were reported. Our Neural Network topography was the following: The models had an input layer, a single hidden layer and an output layer. The amount of neurons for the input layer was equal to the size of the feature vector. For the hidden layer, models trained with both subsets of data had 55 neurons while models trained with a single subset of data had 30 neurons. All models had single output neuron.

The SVM kernel, used a Gaussian radial basis function $K\left(x;y\right)=exp(-1/\sigma^{2}{\left(x-y\right)}^{2})$ because of its popularity for SVM classification problems.

In the Random Forest model the only parameter tweaked was the number of trees, which was raised from its 10 default up to 1000.

### 3.2. Evaluation

To evaluate the robustness of each model we used accuracy, precision, recall and $f_{1}$ scores which are defined as follows:

(4) $$Accurracy=\frac{t_{p}+t_{n}}{t_{p}+t_{n}+f_{p}+f_{n}}$$

(5) $$Precision=\frac{t_{p}}{t_{p}+f_{p}}$$

(6) $$Recall=\frac{t_{p}}{t_{p}+f_{n}}$$

(7) $$f_{1}=2\frac{precision*recall}{precision+recall},$$

where,

$t_{p}$ = Number of true positive values
$t_{n}$ = Number of true negative values
$f_{p}$ = Number of false positive values
$f_{p}$ = Number of false negative values.

Accuracy measures the ratio of all testing samples classified correctly, precision is the ratio of relevant classified samples among the total retrieved samples, recall is the ratio of relevant classified samples among the total amount of relevant samples and $F_{1}$ score is the harmonic average of the precision and recall. Precision was considered the most important score, as it implies how many times we were correct in our prediction which would determine what type of market order a strategy would create.

Confidence intervals where obtained by applying KFold cross validation, with a *K* value equal to 5, which was selected after executing the validation exercises multiple times with new random splits.

### 3.3. Results

Table 3, Table 4, Table 5 and Table 6 show the scores of each of our models applied to the previously defined cryptocurrencies for predicting the market movement of the next day. We include a random and a majority classifier for each exercise. The data sets are available in a public repository listed in the data accessibility section. Data sets contain all the market and *Twitter* data after being processed. Raw tweets were not included because of size limitations.

As we can see in Table 3, MLP was the best performing model for Bitcoin. Having an accuracy of over 0.72 and a precision of 0.76, this model is better than random by a large margin. Both SVM and RF also managed to beat random when using Market data. Twitter data by itself could not be used to predict the market movement in any model, and its inclusion appeared to worsen the performance of the SVM and RF models. However it improved the precision in the MLP model slightly.

For Ethereum the best performing model was MLP as shown in Table 4. No model was able to perform significantly better than random. MLP was the only model that was able to obtain a slight edge in precision against random by including both market and Twitter data. Neither Twitter data nor market data by themselves were able to predict the Ethereum market movements.

In Table 5 we can see how for Ripple, MLP was again the best performing model obtaining a 0.64 accuracy and a 0.68 precision score beating random by a large margin. SVM also beat random by a small margin when using only Twitter data. RF did not manage to beat random. Twitter data was able to beat random by itself when using the SVM model with 0.53 accuracy and 0.6 precision scores.

Table 6 shows how SVM was the best performing model for Litecoin, obtaining a 0.66 accuracy and a 0.8 precision score. RF performed slightly better than MLP when using both Twitter data and market data. All models were able to beat random. Twitter data was able to predict the market by itself when using the MLP and RF models.

## 4. Discussion

Our results show that for the Bitcoin, Ethereum, Ripple and Litecoin markets there is at least one model that can predict market movements by beating random in precision scores. This prediction is limited to the direction of the market and does not include the magnitude or duration of such market movement. Bitcoin’s best model was a MLP which using both Twitter and market data, obtained scores of 0.72 accuracy and 0.74 precision. Ethereum’s best model was also a MLP that used both Twitter and market data to obtain 0.44 accuracy and 0.56 precision scores, which was not significantly better than random. In Ripple, once again, the best model was an MLP that only used market data, obtaining 0.64 accuracy and 0.68 precision scores. Litecoin was the only cryptocurrency where the SVM model performed the best, using both Twitter and market data it obtained 0.66 accuracy and 0.8 precision scores.

With the highest precision score, Litecoin was the most predictable market, followed by Bitcoin and Ripple. Only the Ethereum market had an accuracy score of under 0.50. MLP was the most successful model, managing to successfully predict market movement prices in all cryptocurrencies while outperforming the other models in three out of four cases. SVM was successful in predicting the markets for Bitcoin, Ripple and Litecoin while failing to predict Ethereum’s. RF was able to predict the Bitcoin and the Litecoin markets.

It is interesting to see how different the results are among the different cryptocurrencies. The best results were obtained for Bitcoin which was expected, falling along the claims of other studies. For Ethereum the accuracy is low for all methods, it is unknown if this could be caused by a market mood or if there is something inherently different in this market. Discovering the root cause of this behavior would be the subject of another study. Such study would require tracking over a longer period of time all relevant factors that would influence market moods. For Ripple and Litecoin we also observed anomalies in the Twitter results which have a better accuracy than market data or both.

We hypothesise that there can be multiple causes for this anomaly. First the Ripple and Litecoin communities have a significantly smaller size, which could mean that Twitter activity would be have a smaller volume but could be more significant. As previously stated, not all social media messages are of equal impact [36]. Secondly we acknowledge that the usage of both the market and Twitter data as a single feature vector could have been a poor design. In hindsight, separating the models and then using a voting mechanism could have yielded better results. Comparing the followed methodology against the proposed one of having separate models, even mixing different types of models, for each different stream of data would be interesting to see.

These results also make it possible to observe how the usage of exclusively Twitter data can be used by itself to predict the Ripple and the Litecoin markets, but it is not superior to the utilization of exclusively market data. The use of both Twitter data and Market data may bring slight improvements in scores, however in other cases it may also cause a reduction in the model performance. When using SVM models, it is theorized that this reduction in performance could be caused by the utilization of a single kernel function for different types of data. It is unknown why this problem occurs with NN and RF models and such question exceeds the scope of this study.

## 5. Conclusions

In this paper, we proved that it is possible to predict the direction of price movements for the emerging cryptocurrency market utilizing machine learning and sentimental analysis, techniques that had been previously utilized for Bitcoin. We evaluated and compared the performance of three prediction models: MLPs, SVMs and RFs for Bitcoin, Ethereum, Ripple and Litecoin using Twitter data, market data or both.

We also demonstrated how cryptocurrency markets, can be a field with a lot of potential for research in financial time series problems because of their high data availability and accessibility.

There is plenty of further work to be done in this area. The application of sentiment analysis for collecting *social signals* could be enhanced by improving the quality of the content, and the number of sources from where such content is gathered. Quality could be bettered by eliminating duplication and filtering content from bots or advertising. As proven before, using content from other social networks such as Reddit or Facebook [37] is possible and would likely be beneficial.

Another area of opportunity would be the usage of more specialized models that have different types of attention mechanisms such as long short-term memory networks (LSTM) and temporal multi layer perceptrons (T-MLP), Recent work shown that the predictability of LSTMs is significantly higher when compared to the generalized regression neural architecture [15]. These kinds of networks may be able to pick the inherent “moods” of the market, and adapt according to it.

We also encourage the use of separate models for Twitter and market data in order to improve models accuracy and precision scores. Finally proving whether these predictive models can be used for creating trading strategies would be interesting.

## Figures and Tables

**Table 1:** Description of collected Tweets.

Cryptocurrency	Collected Tweets	Total Percentage
Bitcoin	13,096,598	63%
Ethereum	5,366,126	25.81%
Ripple	1,143,634	5.5%
Litecoin	1,183,214	5.69%

**Table 2:** Price movements in days for each cryptocurrency.

Cryptocurrency	Price Increased	Price Decreased
Bitcoin	28	32
Ethereum	28	32
Ripple	23	37
Litecoin	29	31

**Table 3:** Results of applying multi-layer perceptron (MLP), support vector machine (SVM) and random forest (RF) using Twitter data, market data or both for predicting daily market movements for Bitcoin.

Model	Accuracy (95% CI)	Precision	Recall	*F*_1_ Score
MLP Twitter	0.39 (±0.02)	0.38	0.39	0.38
MLP Market	0.72 (±0.03)	0.74	0.72	0.71
MLP Twitter and Market	0.72 (±0.06)	0.76	0.72	0.72
SVM Twitter	0.50 (±0.03)	0.29	0.50	0.37
SVM Market	0.55 (±0.03)	0.53	0.56	0.47
SVM Twitter and Market	0.55 (±0.03)	0.31	0.56	0.40
RF Twitter	0.44 (±0.04)	0.50	0.80	0.62
RF Market	0.61 (±0.04)	0.67	0.25	0.36
RF Twitter and Market	0.44 (±0.04)	0.28	0.44	0.34
Random	0.50 (±0.28)	0.49	0.50	0.50
Majority	0.55 (±0.0)	0.31	0.56	0.40

**Table 4:** Results of applying MLP, SVM and RF using Twitter data, market data or both for predicting daily market movements for Ethereum.

Model	Accuracy (95% CI)	Precision	Recall	*F*_1_ Score
MLP Twitter	0.39 (±0.02)	0.44	0.39	0.38
MLP Market	0.44 (±0.02)	0.44	0.39	0.35
MLP Twitter and Market	0.44 (±0.03)	0.56	0.44	0.39
SVM Twitter	0.39 (±0.03)	0.15	0.39	0.22
SVM Market	0.39 (±0.03)	0.15	0.39	0.22
SVM Twitter and Market	0.39 (±0.03)	0.15	0.39	0.22
RF Twitter	0.33 (±0.03)	0.14	0.33	0.19
RF Market	0.28 (±0.03)	0.12	0.28	0.17
RF Twitter and Market	0.39 (±0.03)	0.15	0.39	0.22
Random	0.50 (±0.28)	0.54	0.50	0.49
Majority	0.61 (±0.0)	0.37	0.61	0.46

**Table 5:** Results of applying MLP, SVM and RF using Twitter data, market data or both for predicting daily market movements for Ripple.

Model	Accuracy (95% CI)	Precision	Recall	*F*_1_ Score
MLP Twitter	0.54 (±0.03)	0.50	0.50	0.50
MLP Market	0.64 (±0.04)	0.68	0.67	0.66
MLP Twitter and Market	0.56 (±0.02)	0.56	0.56	0.55
SVM Twitter	0.53 (±0.04)	0.60	0.56	0.50
SVM Market	0.50 (±0.04)	0.50	0.50	0.41
SVM Twitter and Market	0.50 (±0.04)	0.25	0.50	0.33
RF Twitter	0.39 (±0.03)	0.39	0.39	0.39
RF Market	0.50 (±0.03)	0.50	0.50	0.41
RF Twitter and Market	0.44 (±0.03)	0.44	0.44	0.44
Random	0.50 (±0.28)	0.50	0.50	0.49
Majority	0.50 (±0.0)	0.25	0.50	0.33

**Table 6:** Results of applying MLP, SVM and RF using Twitter data, market data or both for predicting daily market movements for Litecoin.

Model	Accuracy (95% CI)	Precision	Recall	*F*_1_ Score
MLP Twitter	0.59 (±0.05)	0.61	0.61	0.61
MLP Market	0.61 (±0.04)	0.78	0.61	0.54
MLP Twitter and Market	0.61 (±0.04)	0.62	0.61	0.60
SVM Twitter	0.52 (±0.04)	0.50	0.50	0.41
SVM Market	0.52 (±0.04)	0.25	0.50	0.33
SVM Twitter and Market	0.66 (±0.04)	0.80	0.67	0.62
RF Twitter	0.50 (±0.03)	0.50	0.50	0.49
RF Market	0.50 (±0.03)	0.50	0.50	0.49
RF Twitter and Market	0.61 (±0.03)	0.66	0.61	0.58
Random	0.50 (±0.28)	0.50	0.50	0.50
Majority	0.50 (±0.0)	0.25	0.50	0.33

## Data Availability

Data used for this article is publicly available either though the corresponding Application Programming Interface or it is available alongside the required scripts to reproduce these results at https://github.com/vanclief/algo-trading-crypto.

## References

[1] Ferreira M., Rodrigues S., Reis C.I., Maximiano M. (2018). Blockchain: A Tale of Two Applications. Appl. Sci..

[2] Trabelsi N. (2018). Are There Any Volatility Spill-Over Effects among Cryptocurrencies and Widely Traded Asset Classes?. J. Risk Financ. Manag..

[3] Corelli A. (2018). Cryptocurrencies and Exchange Rates: A Relationship and Causality Analysis. Risks.

[4] Cocco L., Tonelli R., Marchesi M. (2019). An Agent Based Model to Analyze the Bitcoin Mining Activity and a Comparison with the Gold Mining Industry. Future Internet.

[5] Memon R.A., Li J.P., Ahmed J. (2019). Simulation Model for Blockchain Systems Using Queuing Theory. Electronics.

[6] Hölbl M., Kompara M., Kamišalić A., Nemec Zlatolas L. (2018). A Systematic Review of the Use of Blockchain in Healthcare. Symmetry.

[7] Fischer T.G., Krauss C., Deinert A. (2019). Statistical Arbitrage in Cryptocurrency Markets. J. Risk Financ. Manag..

[8] Garcia D., Schweitzer F. (2015). Social signals and algorithmic trading of Bitcoin. R. Soc. Open Sci..

[9] Bollen J., Maoa H., Zeng X. (2010). Twitter mood predicts the stock market. J. Comput. Sci..

[10] Li Q., Wang T., Li P., Gong Q., Chen Y. (2014). The effects of news and public mood on stock movements. Inf. Sci..

[11] Chen C.-H., Hafner C.M. (2019). Sentiment-Induced Bubbles in the Cryptocurrency Market. J. Risk Financ. Manag..

[12] Bekiros S., Gupta R., Kyei C. (2016). A non-linear approach for predicting stock returns and volatility with the use of investor sentiment indices. Appl. Econ..

[13] Lahmiri S., Bekiros S. (2018). Chaos, randomness and multi-fractality in Bitcoin market. Chaos Solitons Fractals.

[14] Chen A., Leung M., Daouk H. (2014). Application of Neural Networks to an Emerging Financial Market: Forecasting and Trading the Taiwan Stock Index. Comput. Oper. Res..

[15] Lahmiri S., Bekiros S. (2019). Cryptocurrency forecasting with deep learning chaotic neural networks. Chaos Solitons Fractals.

[16] Bekiros S.D., Georgoutsos D.A. (2008). Direction-of-change forecasting using a volatility-based recurrent neural network. J. Forecast..

[17] Saad E., Prokhorov D., Wunsch D. Advanced neural network training methods for low false alarm stock trend prediction. Proceedings of the IEEE International Conference on Neural Networks (ICNN96).

[18] Tsaih R., Hsu Y., Lai C.C. (1998). Forecasting S&P 500 stock index futures with a hybrid AI system. Decis. Support Syst..

[19] Kohara K., Ishikawa T., Fukuhara Y., Nakamura Y. (1997). Stock price prediction using prior knowledge and neural networks. Int. Syst. Account. Financ. Manag..

[20] Baestaens D.E., van den Bergh W.M. (1995). Tracking the Amsterdam stock index using neural networks. Neural Netw. Cap. Mark..

[21] Tsibouris G., Zeidenberg M. Back propagation as a test of the efficient markets hypothesis. Proceedings of the Twenty-Fifth Hawaii International Conference on System Sciences.

[22] Refenes A.-P., Zapranis A.D., Francis G. (1995). Modeling stock returns in the framework of APT: A comparative study with regression models. Neural Netw. Cap. Mark..

[23] Cao L., Tay F.E. (2001). Financial forecasting using support vector machines. Neural Comput. Appl..

[24] Cao L., Tay F.E. (2001). Application of support vector machines in financial time series forecasting. Omega.

[25] Huang W., Nakamori Y., Wang S.Y. (2005). Forecasting stock market movement direction with support vector machine. Comput. Oper. Res..

[26] Chen W., Shih J. (2006). Comparison of support-vector machines and back propagation neural networks in forecasting the six major Asian stock markets. Int. J. Electron. Financ..

[27] Patel J., Shah S., Thakkar P., Kotecha K. (2015). Predicting stock and stock price index movement using Trend Deterministic Data Preparation and machine learning techniques. Expert Syst. Appl..

[28] Suryoday B., Saibal K., Snehanshu S., Luckyson K., Sudeepa R. (2019). Predicting the direction of stock market prices using tree-based classifiers. N. Am. Econ. Financ..

[29] Bordino I., Battiston S., Caldarelli G., Cristelli M., Ukkonen A., Weber I. (2012). Web search queries can predict stock market volumes. PLoS ONE.

[30] Schoen H., Gayo-Avello D., Metaxas P.T., Mustafaraj E., Strohmaier M., Gloor P. (2013). The power of prediction with social media. Internet Res..

[31] Kim Y.B., Kim J.G., Kim W., Im J.H., Kim T.H., Kang S.J., Kim C.H. (2016). Predicting Fluctuations in Cryptocurrency Transactions Based on User Comments and Replies. PLoS ONE.

[32] Phillips R.C., Gorse D. (2018). Cryptocurrency price drivers: Wavelet coherence analysis revisited. PLoS ONE.

[33] Hutto C.J., Gilbert E. VADER: A Parsimonious Rule-based Model for Sentiment Analysis of Social Media Text. Proceedings of the Eighth international AAAI Conference on Weblogs and Social Media.

[34] Ribeiro F., Araújo M., Gonçalves P., Gonçalves M., Benevenuto F. (2016). SentiBench—A benchmark comparison of state-of-the-practice sentiment analysis methods. EPJ Data Sci..

[35] Haganm M., Demuth H., Hudson M., Orlando-De-Jesús B. (2014). Neural Network Design.

[36] Mai F., Shan Z., Bai Q., Wang X., Chiang R. (2018). How Does Social Media Impact Bitcoin Value? A Test of the Silent Majority Hypothesis. Manag. Inf. Syst..

[37] Garcia D., Tessone C., Mavrodiev P., Perony N. (2014). The digital traces of bubbles: Feedback cycles between socio-economic signals in the Bitcoin economy. J. R. Soc. Interface.

